# Evaluation of N95 respirator and surgical mask application on intraocular pressure and superficial retinal vessel density in healthy subjects

**DOI:** 10.3389/fmed.2026.1764952

**Published:** 2026-04-10

**Authors:** Kailin Xiao, Ruoshi Cai, Li Tan, Xiaoling Xie, Kunliang Qiu, Tsz Kin Ng, Chukai Huang

**Affiliations:** 1 Joint Shantou International Eye Center of Shantou University and the Chinese University of Hong Kong, Shantou, Guangdong, China; 2 Shantou University Medical College, Shantou, Guangdong, China; 3 Department of Ophthalmology and Visual Sciences, The Chinese University of Hong Kong, Hong Kong, Hong Kong SAR, China

**Keywords:** intraocular pressure, N95 respirator, OCT angiography, retinal vessel density, surgical mask

## Abstract

**Purpose:**

To evaluate the changes in intraocular pressure (IOP) and superficial retinal vessel density after short-term application of N95 respirators or regular surgical masks in healthy subjects.

**Methods:**

Twenty-seven healthy subjects were recruited, and each received three interventions: without wearing a mask, wearing a regular surgical mask, and wearing an N95 respirator for 30 min. With a one-hour washout interval between mask conditions. IOP and superficial retinal vessel density were measured immediately before and after each intervention.

**Results:**

There was no significant difference in IOP in either eye (analyzed on a per-eye basis, *n* = 54) after wearing a surgical mask or N95 respirator compared to the no-mask condition. No significant difference was found in superficial retinal vessel density of the inferior, superior, nasal, and temporal areas within a diameter of 3 mm centered on the macular fovea, nor in the foveal avascular zone within a diameter of 1 mm centered on the macular fovea of both eyes, across all three conditions.

**Conclusion:**

Short-term (30-min) application of an N95 respirator or surgical mask does not significantly affect IOP or superficial retinal vessel density in healthy young adults. These findings provide preliminary evidence regarding the ocular safety of short-term mask use in this population.

## 1 Introduction

Mask-wearing has been widely adopted as a crucial measure for preventing virus transmission during the COVID-19 pandemic (1, 2). Previous research has shown that respiratory patterns, respiratory rate, and breathing maneuvers can influence IOP fluctuations (3–5). For instance, breath holding and the Valsalva maneuver—forced exhalation against a closed airway—can lead to temporary IOP elevations by increasing intrathoracic pressure and impeding venous drainage from the eye. Furthermore, cardiovascular circulation can cause transient or permanent changes in ocular perfusion pressure, raising episcleral pressure and choroidal vessels’ engorgement (3, 6), resulting in visual function impairment due to the elevation of IOP.

Previous studies have demonstrated that different breathing patterns during physical resistance exercises may cause changes in IOP, with the Valsalva maneuver resulting in an increase in IOP (4, 7). Additionally, variations in oxygen concentration can cause changes in ocular biological characteristics, which affect IOP (8, 9). Medical masks, including surgical masks and N95 respirators, are designed to block droplet transmission while maintaining respiratory patency. However, wearing masks may lead to alterations in respiratory frequency and amplitude, given the varied ventilate resistance of different masks. Surgical masks generally have ventilate resistance below 45 pascal (Pa), while N95 masks have approximately 300 Pa (10). While the physiological effects of extreme respiratory maneuvers (e.g., Valsalva) on IOP are well-established, it remains unknown whether the modest increase in respiratory resistance associated with routine mask use is sufficient to induce clinically meaningful IOP changes. This distinction is important because the magnitude of pressure change generated by mask-related resistance (∼300 Pa) is orders of magnitude smaller than that produced by the Valsalva maneuver, which can generate intrathoracic pressures of tens to hundreds of millimeters of mercury.

Given the widespread and prolonged use of masks in clinical and community settings, understanding their potential ocular effects is clinically relevant. Therefore, we conducted a study to evaluate IOP and superficial retinal vessel density changes before and after wearing surgical masks or N95 respirators in healthy participants and compared to that without mask application.

## 2 Materials and methods

### 2.1 Study design and participants

**Figure 1:**
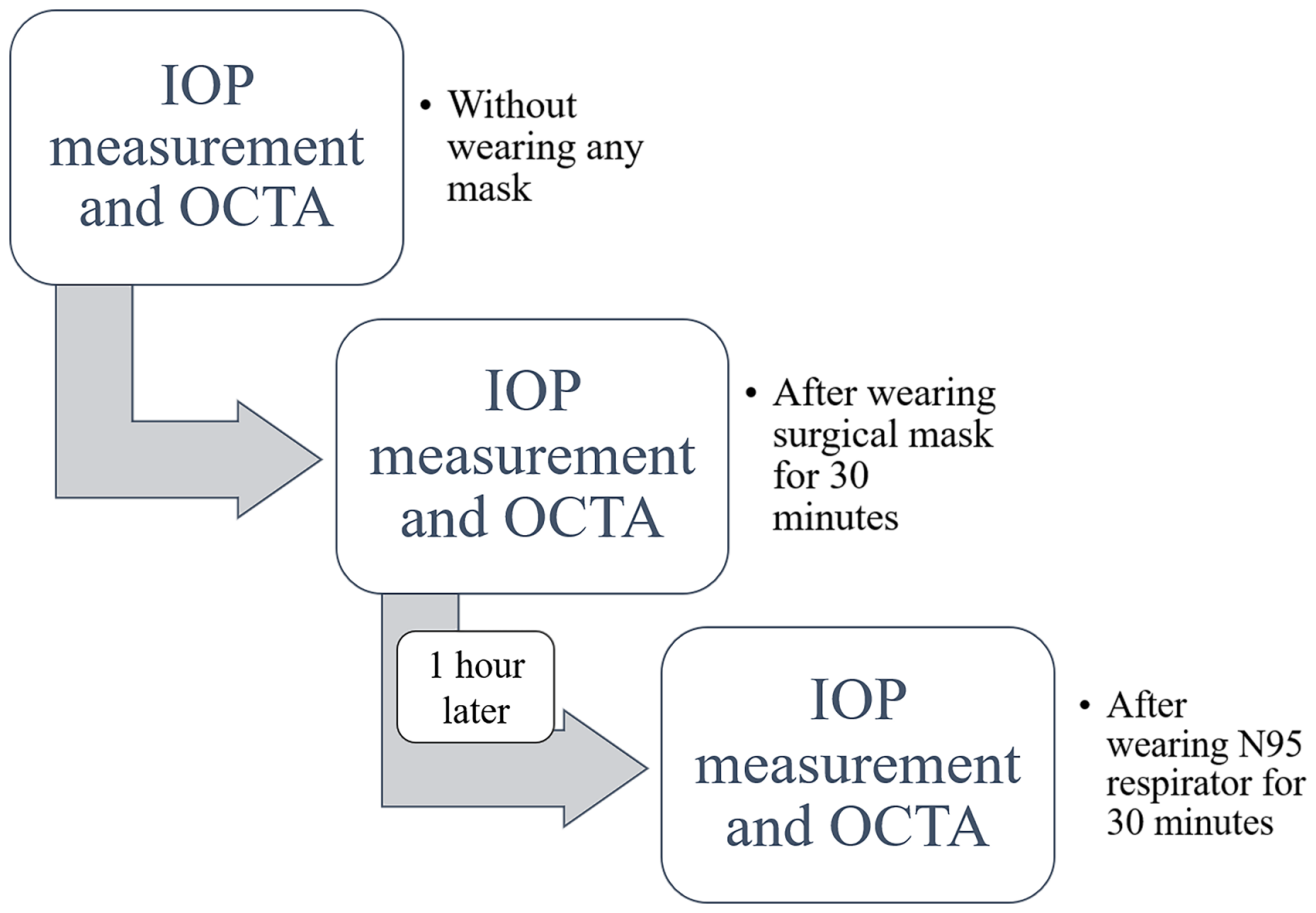
Study protocol flowchart. This diagram illustrates the sequence of interventions and measurements. All study participants underwent a baseline assessment without a mask, followed by a 30-min period wearing a regular surgical mask, and finally a 30-min period wearing an N95 respirator. Intraocular pressure (IOP) and optical coherence tomography-angiography (OCTA) scans were performed immediately before and after each 30-min intervention. A one-hour washout period without a mask was implemented between the surgical mask and N95 respirator interventions.

**Figure 2:**
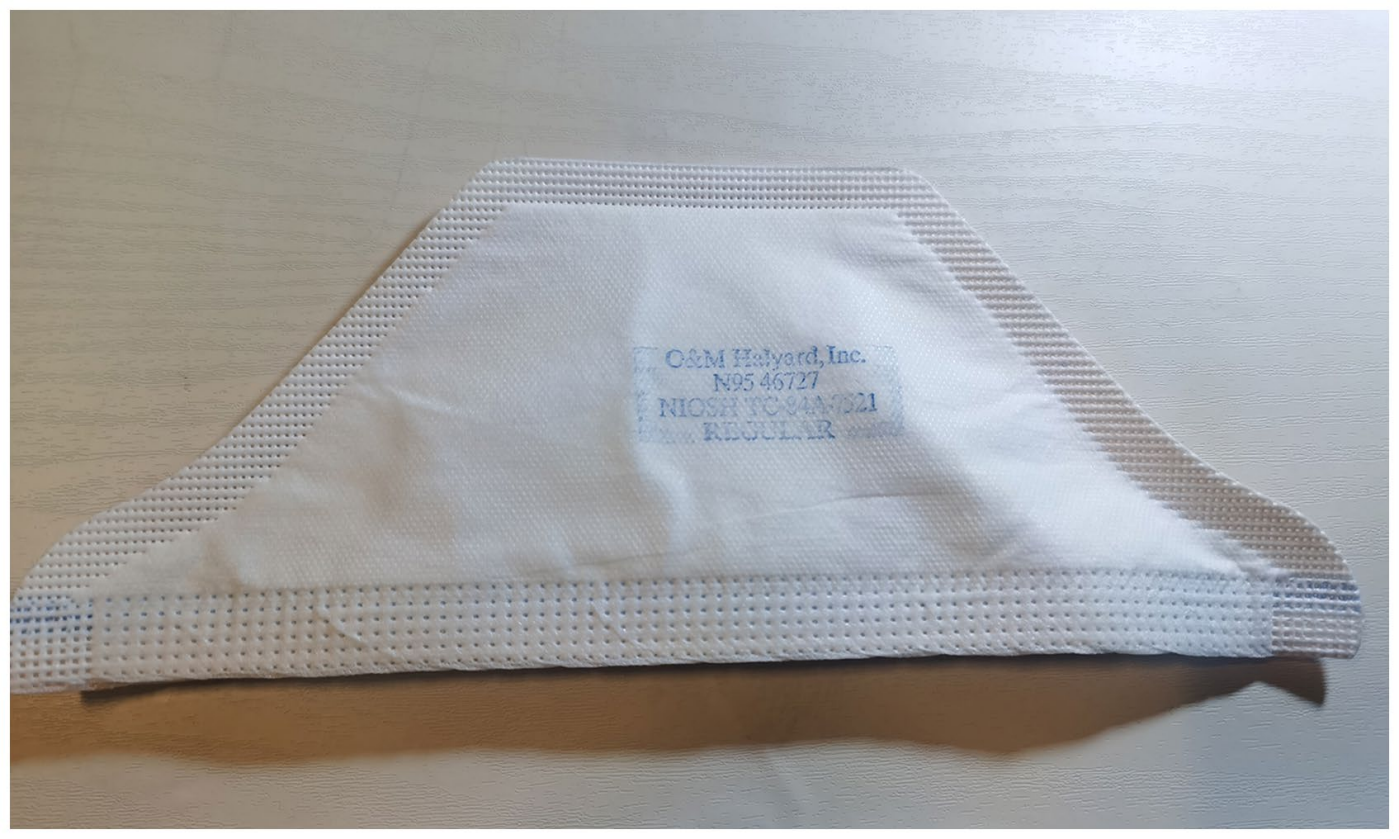
The N95 respirator used in the study. A disposable N95 respirator (Model 46727; Medicom, Canada) is shown. This particulate respirator is certified by the National Institute for Occupational Safety and Health (NIOSH).

**Figure 3:**
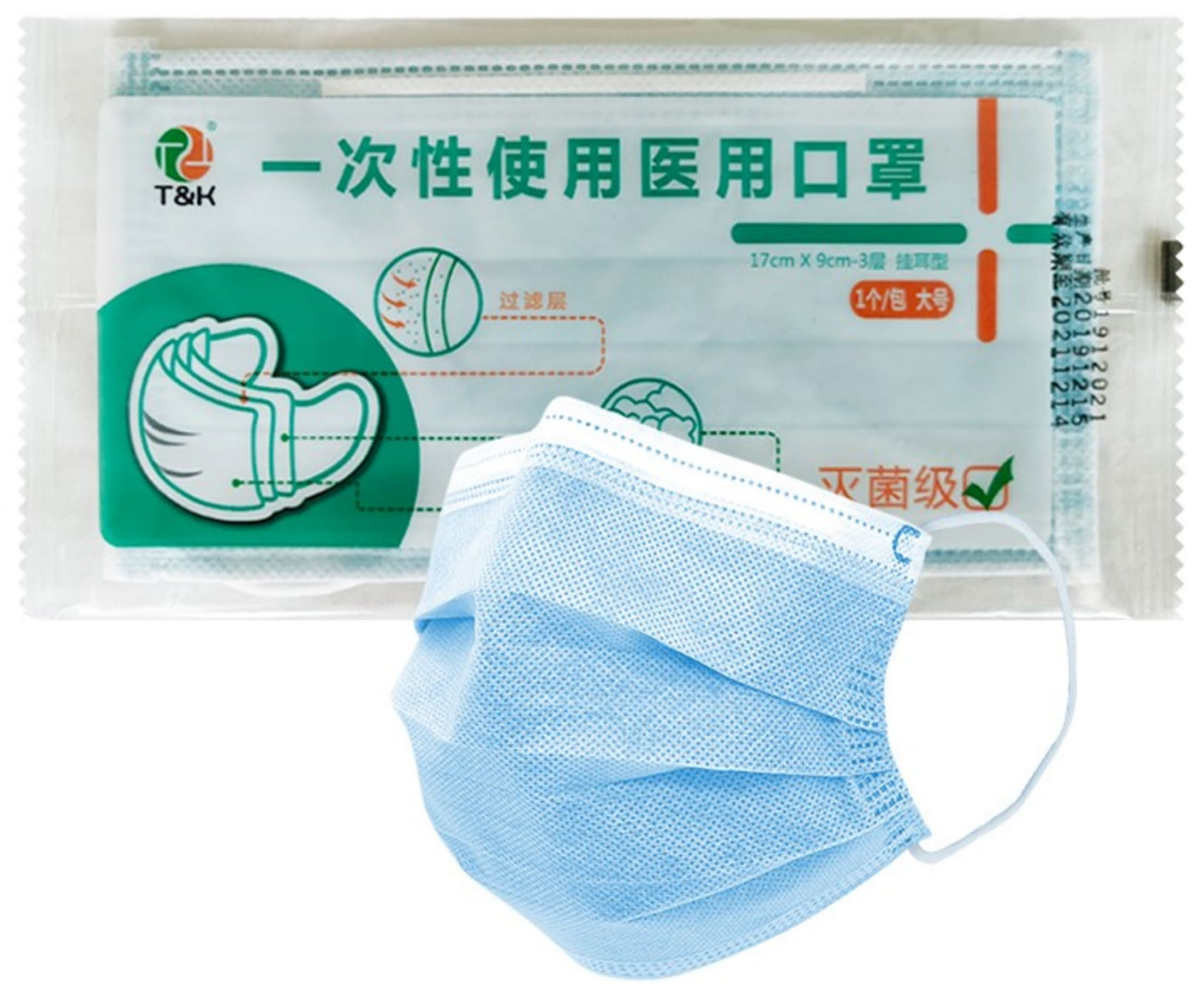
The surgical mask used in the study. A disposable regular surgical mask (T&K, China) is shown. The mask dimensions are 17 cm × 9 cm, with a 3-ply construction and ear-loop design. It was certified by the National Medical Products Administration, China.

We recruited 27 healthy participants (9 males and 18 females) at the Joint Shantou International Eye Center of Shantou University and the Chinese University of Hong Kong. Inclusion criteria were age ≥18 years, no history of ocular disease or surgery, and no systemic conditions known to affect IOP or retinal circulation. Each participant was instructed to follow a study protocol, which involved three stages in a fixed sequence: (1) no mask wearing (baseline control), (2) wearing a surgical mask for 30 min, and (3) wearing an N95 respirator for 30 min, with a one-hour washout period without mask wear between the surgical mask and N95 respirator conditions (Figure 1). The N95 respirator, a disposable protective mask type (Medicom, Canada; Figure 2), was certified by the National Institute for Occupational Safety and Health (NIOSH). Conversely, the disposable surgical facemasks (T&K, China; Figure 3) were certified by the National Medical Products Administration, China. All measurements were performed immediately before and immediately after each 30-min intervention, with participants resting quietly in a seated position during the mask-wear periods. Our study protocol was approved by the Ethics Committee for Human Research at the Joint Shantou International Eye Center of Shantou University and the Chinese University of Hong Kong (Approval Number: EC20200512(4)-P02) in line with the principles of the Declaration of Helsinki. All study participants provided written informed consent after receiving an explanation on the study’s nature and possible consequences.

### 2.2 Ophthalmic examinations

All study participants underwent complete ophthalmic examinations, including slit lamp and fundus examination, to rule out any ocular diseases. The IOP was measured by Goldmann tonometer between 3:00–6:00 p.m. in June for all participants to minimize diurnal variation. Two measurements were taken and averaged if the difference between the two measurements did not exceed 0.5 mmHg. If the difference surpassed 0.5 mmHg, the measurement was repeated until the difference did not exceed this value. For both eyes, optical coherence tomography-angiography (OCTA) scans were taken, using a DRI-OCT Triton (Topcon Corporation, Tokyo, Japan). The scans included a 7 × 7 mm B-scan centered on the macular fovea, followed by a 6 × 6 mm OCTA centered on the same area. Superficial retinal vessel density was analyzed using Topcon ImageNet^®^ software (Topcon Corporation, Tokyo, Japan). The software automatically segmented the superficial retinal layer from 2.6 μm below the inner limiting membrane to the inner plexiform layer (15.6 μm below the inner limiting membrane). Vessel density was calculated for the foveal avascular zone (FAZ) within a 1 mm diameter centered on the fovea, and for four parafoveal quadrants (inferior, superior, nasal, temporal) within a 3 mm diameter centered on the fovea. All IOP and OCTA measurements were obtained immediately after mask removal (within 1 min) to capture any potential acute effects while avoiding measurement artifacts during mask wear. The examinations were carried out in the following order: (1) baseline without wearing a mask for 30 min, (2) wearing a surgical mask for 30 min, and (3) wearing an N95 respirator for 30 min. Participants were not wearing a mask for an hour after the initial surgical mask use before transitioning to the N95 respirator. The IOP values of both eyes and superficial retinal vessel density (SRVD) in the central avascular zone, the inferior, superior, nasal, and temporal areas within a 3 mm diameter centered on the macular fovea in both eyes were recorded.

### 2.3 Statistical analysis

The data for each variable followed a normal distribution (Shapiro–Wilk test, *p* > 0.05). The mean and standard deviation (SD) were used to describe the IOP and retinal vascular density data. Repeated measures ANOVA was used to compare the IOP and retinal vascular density before wearing a mask, after wearing a surgical mask, and after wearing an N95 respirator. All analyses were conducted on a per-eye basis (*n* = 54 eyes), and this approach was explicitly maintained throughout the statistical analysis. IBM SPSS Statistics 25 (SPSS Inc., Chicago, IL) was used for all statistical analyses. Statistical significance was defined as *p* < 0.05 (two-tailed).

## 3 Results

### 3.1 Participant characteristics

Twenty-seven healthy participants were enrolled in our study, including nine males and 18 females, averaging 24.3 ± 1.3 years in age (range, 22–28 years). All participants completed all three experimental conditions without adverse events.

### 3.2 Intraocular pressure changes after mask wearing

**Figure 4:**
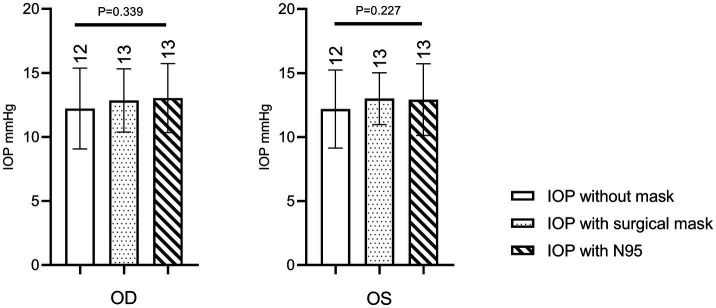
Repeated measures ANOVA revealed no significant difference in the IOP of either eye between wearing the masks and not wearing any masks.

Table 1 presents the mean IOP values for both eyes under each condition. As shown in Figure 4, there was no significant change in IOP before, after wearing a regular surgical mask or after wearing an N95 respirator. Before wearing the mask, the mean IOP was 12 ± 3 mmHg for both the right and left eyes. After wearing a surgical mask and an N95 respirator for 30 min, the mean IOP was 13 ± 3 mmHg for both eyes. Repeated measures ANOVA revealed no significant difference in the IOP of either eye between wearing the masks and not wearing any masks (right eye: *p* = 0.339; left eye: *p* = 0.227).

**Table 1:** Intraocular pressure (mmHg) before and after mask wearing.

Condition	Right eye (mean ± SD)	Left eye (mean ± SD)
No mask (baseline)	12 ± 3*	12 ± 3*
After surgical mask (30 min)	13 ± 3*	13 ± 3*
After N95 respirator (30 min)	13 ± 3*	13 ± 3*

^*^Repeated measures ANOVA revealed no significant difference in IOP across the three conditions for either eye (right eye: *p* = 0.339; left eye: *p* = 0.227).

### 3.3 Retinal vessel density changes after mask wearing

**Figure 5:**
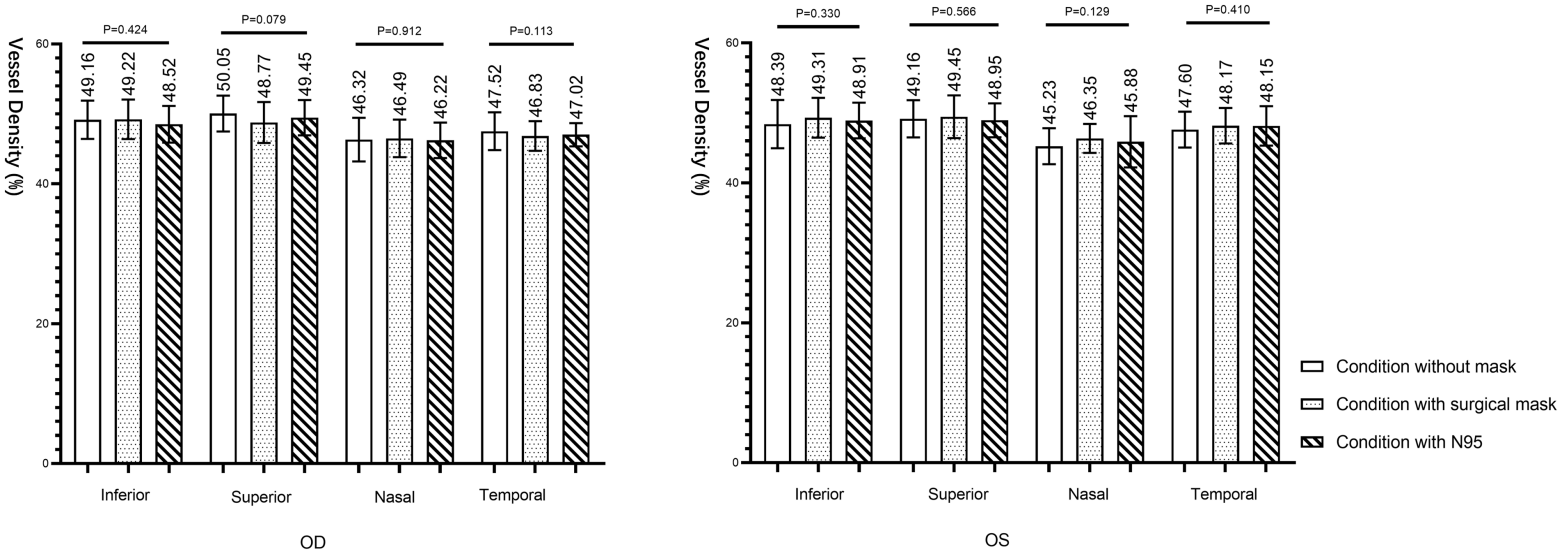
Superficial retinal vessel density in the parafoveal regions.

Table 2 summarizes SRVD in the parafoveal quadrants under each condition. In Figure 5, the vessel densities of the superficial retina were presented within a 3 mm diameter centered on the macular fovea of both eyes. The region extended from 2.6 μm below the inner limiting membrane to the inner plexiform layer (15.6 μm below the inner limiting membrane) in the inferior, superior, nasal, and temporal regions. Repeated measures ANOVA analyzed the masks’ impact on the vessel densities of the study subjects and found no significant difference in the inferior (right eye: *p* = 0.424; left eye: *p* = 0.330), superior (right eye: *p* = 0.079; left eye: *p* = 0.566), nasal (right eye: *p* = 0.912; left eye: *p* = 0.129), and temporal regions (right eye: *p* = 0.113; left eye: *p* = 0.410).

**Table 2:** Superficial retinal vessel density (%) in parafoveal quadrants.

Quadrant	Eye	No mask	Surgical mask	N95 respirator	*p*-value^*^
Inferior	RE	49.16 ± 3.19	49.22 ± 3.52	48.52 ± 3.26	0.424
	LE	48.39 ± 3.72	49.31 ± 4.40	48.91 ± 4.27	0.330
Superior	RE	50.05 ± 2.78	48.77 ± 3.60	49.45 ± 2.97	0.079
	LE	49.16 ± 3.10	49.45 ± 4.21	48.95 ± 3.29	0.566
Nasal	RE	46.32 ± 3.57	46.49 ± 3.52	46.22 ± 3.14	0.912
	LE	45.23 ± 3.43	46.35 ± 3.81	45.88 ± 3.71	0.129
Temporal	RE	47.52 ± 3.41	46.83 ± 3.15	47.02 ± 2.87	0.113
	LE	47.60 ± 3.67	48.17 ± 3.94	48.15 ± 3.75	0.410

^*^Repeated measures ANOVA. RE, right eye; LE, left eye.

**Figure 6:**
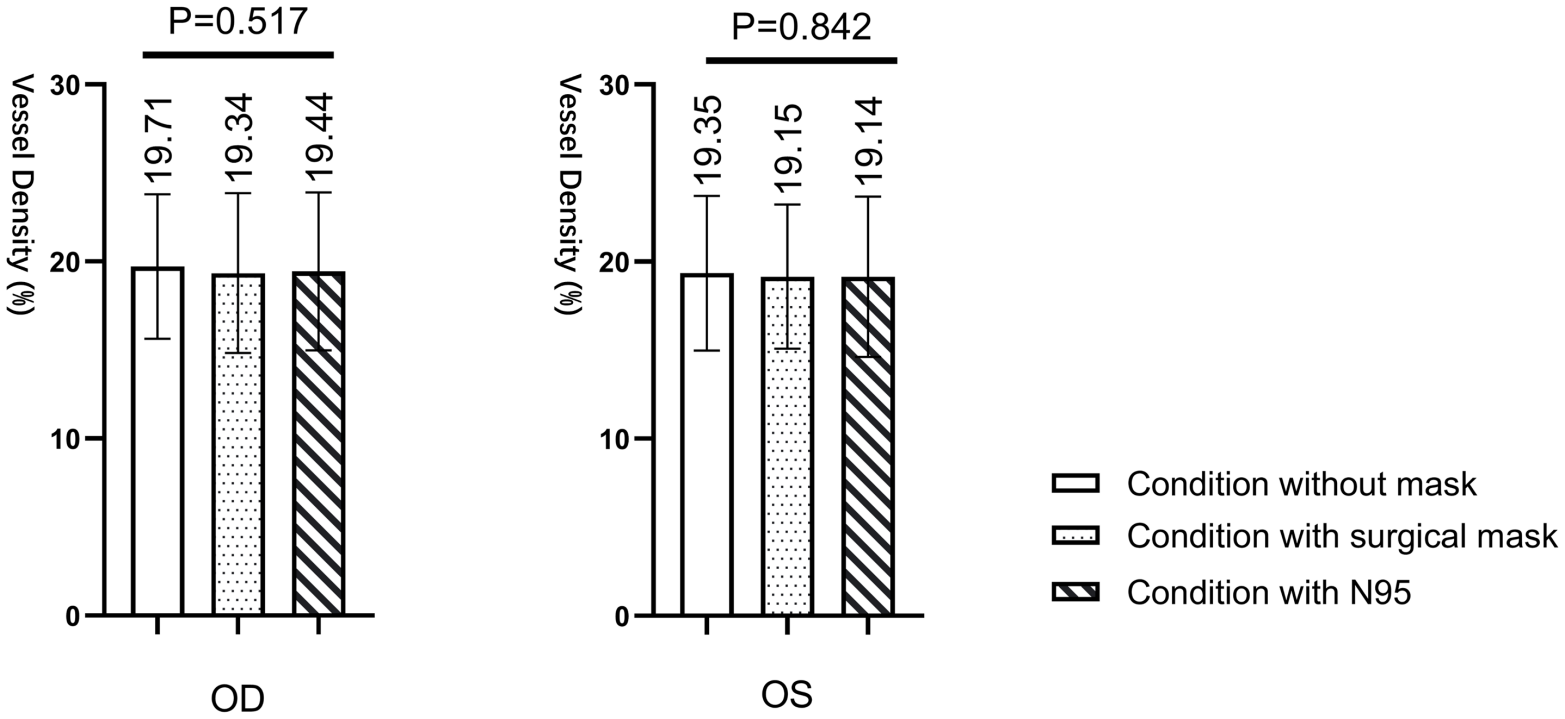
Vessel density in the foveal avascular zone.

Figure 6 illustrated the mean vessel densities of the foveal avascular zone within a 1 mm diameter centered on the macular fovea of both eyes. Table 3 presents vessel density in the foveal avascular zone. The superficial retina region analyzed ranged from 2.6 μm below the inner limiting membrane to the inner plexiform layer (15.6 μm below the inner limiting membrane). The findings from the repeated measures ANOVA indicated that wearing masks had no significant impact on the vessel density of the foveal avascular zone in the study subjects (right eye: *p* = 0.517; left eye: *p* = 0.842).

**Table 3:** Vessel density (%) in the foveal avascular zone.

Eye	No mask	Surgical mask	N95 respirator	*p*-value^*^
Right eye	19.71 ± 4.00	19.34 ± 4.57	19.44 ± 4.48	0.517
Left eye	19.35 ± 4.35	19.15 ± 4.36	19.14 ± 4.47	0.842

^*^Repeated measures ANOVA.

## 4 Discussion

The study’s outcome showed that wearing N95 respirators, surgical masks, or no masks did not significantly affect the IOP of the study subjects. The vascular densities of the superficial retina from 2.6 μm below the inner limiting membrane to the inner plexiform layer in the inferior, superior, nasal, and temporal regions, as well as the foveal avascular zone, were also not significantly impacted by mask-wearing.

### 4.1 Interpretation of findings in context of respiratory physiology

A central consideration in interpreting our negative findings is the magnitude of physiological perturbation induced by mask wear. The ventilatory resistance of N95 respirators (∼300 Pa) (10) is substantially lower than the intrathoracic pressure changes generated by maneuvers known to affect IOP. For example, the Valsalva maneuver—forced exhalation against a closed glottis—can increase intrathoracic pressure by 40–80 mmHg (approximately 5,300–10,600 Pa) or more (7), two orders of magnitude greater than mask-related resistance. This distinction is critical: while previous studies have documented IOP elevations during Valsalva (4, 7), breath holding (5), and unilateral nostril breathing (11), these interventions involve fundamentally different physiological mechanisms and pressure magnitudes compared to routine mask wear.

Our study did not hypothesize that mask wear is equivalent to these extreme respiratory maneuvers. Rather, we sought to determine whether the modest, clinically realistic increase in respiratory resistance associated with mask use could produce any detectable ocular effect. From this perspective, our negative findings are not unexpected—they are consistent with the physiological principle that a threshold of perturbation must be exceeded to evoke measurable changes in IOP or retinal microcirculation. As such, the value of this study lies in providing empirical evidence that routine short-term mask use falls below that threshold in healthy individuals, thereby distinguishing everyday mask wear from the more extreme respiratory challenges studied previously.

### 4.2 Comparison with previous literature

However, the respiratory rhythm of wearing masks differs from normal breathing. According to Lee and Wang (12), rhinomanometry and spirometer techniques showed a 126% increase during inspiration and a 122% increase during expiration in average nasal airflow resistance after wearing N95 masks. These findings suggest that wearing N95 respirators could exacerbate respiratory resistance. Another study conducted by Li et al. (13) found that wearing N95 respirators is less comfortable and can cause different reactions in the human body compared to wearing surgical masks. For example, after wearing N95 respirators for 30 s, air exchange volume decreases by approximately 37%. Notably, the researchers found that wearing N95 respirators may impact microclimate temperature, humidity, skin temperature, respiratory resistance, itch, fatigue, and overall discomfort. These studies establish that mask wear produces measurable respiratory changes. However, our findings suggest that these changes—at least over 30 min—do not translate into alterations in IOP or retinal vessel density.

Studies examining hypoxic conditions have reported variable effects on IOP. At high altitudes, hypobaric hypoxia may cause small but statistically significant IOP changes, but typically requires exposure exceeding 2 h (14). Baertschi et al. (15) suggested that autoregulatory mechanisms maintain IOP stability during mild hypoxia, possibly through adjustments in aqueous humor dynamics. Our 30-min protocol was substantially shorter than the duration required to induce hypoxic ocular effects, and we did not measure blood oxygen saturation or endothelin-1 (ET-1) levels (16, 17). Therefore, while mask wear might theoretically alter inspired gas composition (18), our study cannot address whether longer exposure or different physiological conditions would yield different results.

### 4.3 Methodological considerations

We explicitly analyzed data on a per-eye basis (*n* = 54 eyes) without averaging between eyes, maintaining statistical independence while accounting for bilateral measurements. IOP measurements were obtained immediately after mask removal, ensuring that any acute effects were captured while avoiding artifacts during mask wear. The use of Goldmann tonometry and Topcon OCTA with ImageNet® software represents current clinical standards for IOP assessment and retinal vessel density quantification.

We elected to assess only the superficial retinal capillary plexus in this study. This decision was based on: (1) our primary interest in evaluating IOP and vessel density as independent parameters rather than assuming a mechanistic link between them; (2) evidence that the superficial plexus is more susceptible to perfusion pressure changes than the deeper plexus in some contexts; and (3) technical considerations regarding segmentation reliability in the deeper layers. We acknowledge that the superficial capillary plexus is not an established biomarker for short-term IOP changes, and we did not hypothesize that mask wear would differentially affect specific retinal layers. Future studies may wish to examine both superficial and deep plexuses to provide a more comprehensive assessment of retinal microvascular responses.

### 4.4 Limitations

This study has certain limitations. First, the sample size (*n* = 27 participants, 54 eyes) is relatively modest. While the repeated-measures design enhances statistical efficiency, the study may be underpowered to detect very small physiological effects. We cannot exclude the possibility that mask wear induces subtle changes below our detection threshold, and our negative findings should not be interpreted as definitive evidence of “no effect” at any magnitude.

Second, the study population consisted exclusively of young, healthy adults (mean age 24 years). These results cannot be generalized to older individuals, who may have different baseline IOP, reduced autoregulatory capacity, or age-related vascular changes. Most critically, our findings do not apply to patients with glaucoma or other ocular pathologies, who represent the clinically most relevant population for IOP-related concerns. Glaucoma patients may have impaired autoregulation and may be more susceptible to even minor physiological perturbations.

Third, the mask-wear duration was limited to 30 min per condition. Our results cannot be extrapolated to prolonged or repeated mask use over several hours or days, which is common in healthcare workers and other occupational settings. Studies of healthcare workers wearing masks for >12 h have reported decreased oxygen saturation and visual symptoms (19), suggesting that longer exposure may produce different effects.

Fourth, we did not monitor physiological parameters such as respiratory rate, tidal volume, end-tidal CO₂, or oxygen saturation during mask wear. Such measurements could help characterize the actual physiological burden imposed by different mask types and might reveal compensatory breathing patterns that mitigate potential ocular effects.

Fifth, as noted earlier, we assessed only the superficial retinal capillary plexus. The deep capillary plexus and choroidal circulation may respond differently to systemic physiological changes, and their evaluation would require separate OCTA protocols.

Finally, we did not measure potential confounding factors such as blood pressure, heart rate, or plasma ET-1 levels, which could influence ocular perfusion pressure and retinal microcirculation.

### 4.5 Clinical implications and future directions

Our findings suggest that healthy young adults can wear surgical masks or N95 respirators for short periods without experiencing acute changes in IOP or superficial retinal vessel density. This provides reassurance for routine mask use in this population during clinical encounters, community settings, or short-duration occupational exposure.

However, important questions remain unanswered. Future studies should: (1) evaluate longer durations of mask wear (≥4 h) to model real-world occupational exposure; (2) include older adults and patients with glaucoma or other ocular conditions to determine whether susceptible populations exhibit different responses; (3) incorporate continuous monitoring of respiratory parameters and blood gases to characterize the physiological stress imposed by mask wear; (4) assess both superficial and deep retinal vascular plexuses; and (5) examine whether subjective visual symptoms reported by some mask wearers correlate with any measurable physiological changes.

In conclusion, this study uncovered that the application of protective masks, whether N95 respirators or surgical masks, for 30 min does not affect the IOP or superficial vascular density within the macula in healthy young adults. This is highly relevant in light of the COVID-19 pandemic and the widespread use of masks worldwide. Special attention should be paid to the impact of mask-wearing on IOP and retinal vessel density as it relates specifically to patients with glaucoma. More research, evaluating the usage of masks for longer durations and in patients with ocular disease, is necessary to determine their influence on the ocular microenvironment and related illnesses.

## Data Availability

The raw data supporting the conclusions of this article will be made available by the authors, without undue reservation.
